# Beta-testing the feasibility of a family-based financial incentives smoking cessation intervention with Alaska Native families: Phase 2 of the Aniqsaaq (to breathe) Study

**DOI:** 10.1016/j.conctc.2025.101472

**Published:** 2025-03-17

**Authors:** Brianna N. Tranby, Antonia M. Young, Anne I. Roche, Flora R. Lee, Ashley R. Brown, Barb J. Stillwater, Judith J. Prochaska, Diane K. King, Paul A. Decker, Bijan J. Borah, Michael G. McDonell, Timothy K. Thomas, Christi A. Patten

**Affiliations:** a Department of Psychiatry and Psychology – Mayo Clinic, Rochester, MN, USA; b Research Services, Division of Community Health Services – Alaska, Native Tribal Health Consortium, Anchorage, AK, USA; c Stanford Prevention Research Center, Department of Medicine, Stanford University, Stanford, CA, USA; d Center for Behavioral Health Research and Service – University of Alaska Anchorage, Anchorage, AK, USA; e Division of Clinical Trials and Biostatistics, Department of Quantitative, Health Sciences – Mayo Clinic, Rochester, MN, USA; f Division of Health Delivery Research, Center for the Science of Health Care, Delivery – Mayo Clinic, Rochester, MN, USA; g Department of Community and Behavioral Health, Elson S Floyd, College of Medicine – Washington State University, Spokane, WA, USA; h Research Services – Alaska Native Tribal Health Consortium, Anchorage, AK, USA

**Keywords:** Tobacco, Cessation, American indian, Alaska native, Health disparities, Treatment and intervention, Family-based

## Abstract

**Background:**

Alaska Native and American Indian (ANAI) communities in Alaska have disproportionately high commercial tobacco smoking rates and face barriers to accessing cessation treatment. We beta-tested the feasibility of a remotely delivered, ANAI family-based financial incentive cessation intervention.

**Methods:**

We enrolled 10 “dyads” (i.e., one adult ANAI person who smokes [PWS] and one adult family member of their choice) across Alaska into a culturally tailored 6-month intervention (NCT05209451). PWS completed expired carbon monoxide, salivary cotinine, and self-reported abstinence measures at home during six smoking status check-ins. Both dyad members received financial incentives in escalating amounts for confirmed PWS abstinence. Participants completed baseline and end-of-study surveys.

**Results:**

Eight of the 10 PWS were women, their average age was 45 years (range = 34–57), and mean daily cigarettes smoked was 13 (range = 3–20). Five of the 10 family members were women, and four currently also smoked. Of the 60 check-ins possible among PWS participants, 41 (68 %) were completed; five (50 %) completed all check-ins. Despite minor difficulties with PWS internet connection, lost test kits, and delayed payment receipt, all participants were able to complete check-ins and received payments earned. Five PWS were abstinent at the final 6-month check-in, and two PWS were abstinent at all check-ins. Five PWS completed the end-of-study survey; four reported the intervention was helpful and would recommend it to others.

**Conclusion:**

A family-based financial incentive intervention for smoking cessation with ANAI families appears feasible. Next, a randomized controlled trial will be conducted statewide to evaluate effectiveness and inform future implementation needs.

## 1 Introduction

Alaska Native and American Indian (ANAI) peoples in Alaska have been disproportionately impacted by tobacco-related morbidity and mortality [[1], [2], [3]]. In contrast to other U.S. Indigenous populations, tobacco was not available to ANAI communities in Alaska before contact with Russian fur traders in the 1790s, and is not used in traditional ceremonies [[4], [5]]. Though recent public health initiatives have reduced cigarette smoking prevalence in Alaska, prevalence among ANAI peoples remains more than double that of other races (34 % versus 14 %) [1]. The Healthy Alaskans 2030 State Health Assessment reported that tobacco use and treatment access barriers, especially within remote rural regions, were “very” to “extremely” concerning to Alaska residents [6].

Approximately half of the ANAI population in Alaska live in remote communities not connected to a road system, and only accessible year-round by small aircraft [7]. Thus, virtual interventions offer a promising avenue toward scalability and sustainability, particularly where access to existing evidence-based resources is limited. In addition, Tribal leaders have expressed a need to develop novel smoking cessation treatments that incorporate cultural values and strengths to reduce health disparities among ANAI peoples [[2], [3], [8]].

Several studies, including a recent Cochrane review of 48 randomized-controlled trials (RCTs) have found that financial incentive interventions offering immediate and tangible rewards for smoking abstinence are effective and resulted in long-term cessation However, no published studies have evaluated a financial incentive intervention for smoking cessation among ANAI peoples. Prior individual-based cessation interventions have not demonstrated long-term effectiveness among ANAI peoples in Alaska [[9], [10]] who have recommended incorporating important cultural values of family, intergenerationalism, and community resilience into substance misuse interventions [[11], [12], [13]].

A recent pilot trial among AI adults who smoke (PWS) residing in the Midwest found that culturally-tailored cessation interventions may promote quit attempts and peer dissemination [14]. Prior research among both Indigenous and non-Indigenous populations has also documented familial social support as a motivator to quit smoking [[15], [16], [17]], though this relationship has not yet been evaluated with ANAI families. Thus, a remotely delivered smoking cessation intervention that utilizes both financial incentives and family-based cultural values may be effective among ANAI peoples.

### 1.1 Aniqsaaq study

The previously published *Aniqsaaq* study protocol [18] was co-developed through a community-based participatory research (CBPR) process with Tribal leadership and input from an existing Alaska Native Tribal Health Consortium (ANTHC) Research Consultation Committee (RCC) [[19], [20]]. The study has three phases. In Phase 1, interview participants supported the planned intervention and provided feedback to refine recruitment, messaging, and study materials [21]. In Phase 2, due to the intervention's unique design and potential challenges enrolling and providing financial incentives remotely to family dyads rather than individuals, we conducted a small beta-test to assess feasibility. This paper describes our beta-test with ANAI families in Alaska. In Phase 3, we plan to conduct an RCT to evaluate the effectiveness of the intervention and inform future implementation.

## 2 Materials & methods

The study was approved by the Alaska Area (#2021-10-052) and Mayo Clinic (#22–000513) Institutional Review Boards, and the ANTHC Board of Directors. It was registered on ClinicalTrials.gov (NCT05209451). Recruitment occurred from June–October 2023, and data collection was completed in April 2024.

### 2.1 Participant eligibility and recruitment

We recruited participants as “dyads” (i.e., one ANAI PWS and one adult family member of their choice). Using sample size recommendations for behavioral addictions treatment development [22] and beta-testing of novel digital health interventions [[23], [24]] our accrual target was 10 dyads. Based on prior literature and community feedback during study development [18] “family member” was broadly defined as anyone within or outside the PWS’ household who they considered family, whether ANAI or non-ANAI, and could also smoke. The literature offers little guidance on the effect of a support person's smoking status on cessation among PWS [25]. Based on community feedback [21] and the high prevalence of smoking in ANAI communities, the option to enroll a family member who also smokes could enhance the feasibility. In the subsequent Phase 3 of the study we plan to explore the impact of enrolling with a family member who also smokes on cessation outcomes.

Table 1 lists study eligibility criteria [21] Mayo Clinic and the ANTHC recruited participants statewide in Alaska primarily through paid geotargeted ads and organic posts on Facebook, Instagram, and Twitter. The ads featured culturally-tailored messaging per Phase 1 feedback, and linked to a secure online screening survey. We also contacted waitlisted Phase 1 PWS and family members interested in Phase 2.

**Table 1:** Eligibility criteria for Phase 2 beta-test participants.

Inclusion Criteria	Person who Smokes	Family member
Self-reported AN/AI race and live in Alaska	✓	
≥18 years old	✓	✓
Smoked ≥1 tobacco cigarette over past 7 days^a^	✓	
Smoked an average ≥ 3 cigarettes per day over past 3 months	✓	
Considering or willing to make a quit attempt	✓	
Nominate one adult family member to join them	✓	✓
Willing to complete smoking status check-in tests	✓	
Willing to upload digital photographs of self and test results	✓	
Have access to an internet-connected smart device and mobile device that can receive text messages^b^	✓	✓
Willing to complete a W-9 form	✓	✓
**Exclusion Criteria**		
Used pharmacotherapy or behavioral cessation treatment within past 3 months	✓	
Already enrolled with another family member	✓	✓
Self-reports cannabis use but not willing to quit for study duration^c^	✓	

a Includes cigarillos, roll-your-own cigarettes; excludes cigars, vaping, or e-cigarettes.

b If not, willing to use a loaner iPad with cellular connection provided by the study.

c Smoking cannabis may interfere with iCOquit® monitor breath tests.

### 2.2 Screening and consent

Interested individuals were screened via an online Qualtrics [26] survey or phone. If a family member screened first, they were asked to identify and contact an adult PWS to support in quitting smoking. If a PWS screened first, they were asked to identify and contact a family member to enroll with them, and were encouraged to choose someone they considered safe, trusting, and supportive [21] Ineligible individuals were offered smoking cessation and family wellness resources.

Eligible participants reviewed the informed consent form by phone with a study coordinator to ensure familiarity with the study procedures, and written consent was obtained electronically via DocuSign or by postal mail. To confirm smoking status, consented PWS were mailed an Alere™ iScreen® cotinine oral fluid screening kit [27] and instructions for completing the test and uploading results. Consented PWS were emailed a weblink to a secure Research Electronic Data Capture (REDCap) Survey where they uploaded two photos: 1) face and swab in the mouth while doing the test, and 2) test result. We opted to confirm identity with photos rather than video due to internet bandwidth limitations in Alaska. A positive test reflects saliva cotinine concentration of ≥30 ng per milliliter (ng/mL) indicating current nicotine use. Eligible PWS with a positive test and their consented family member were enrolled in the study as a dyad. Per IRS reporting requirements, and as an additional method of confirming identification, all participants signed a W9 tax identification form.

### 2.3 Baseline survey

All participants completed a baseline survey online in REDCap or over the phone which assessed gender, age, race/ethnicity, community of residence, marital status, education, employment status, and health literacy. PWS answered additional questions about whether or not they live in the same household with their enrolled family member, subsistence lifestyle, cultural identity, average daily cigarettes smoked, and use of other nicotine or tobacco products (e.g., e-cigarettes, Iqmik, commercial chew, etc.). The family member survey included questions on their relationship (e.g., spouse, parent, etc.) and perceived closeness to the PWS [28], cultural identity and subsistence lifestyle if the participant was ANAI, and if current smoking was reported, the average daily cigarettes smoked and use of other nicotine or tobacco products. Participants received a $25 prepaid cash card for completing the baseline survey.

### 2.4 Enrollment

After completing enrollment procedures, study staff confirmed dyad accrual and welcomed participants to the study. Accrued dyads were mailed a package with materials for study participation, printed materials on evidence-based tobacco cessation treatment and family wellness resources, and a handout explaining the timing of PWS check-ins, remuneration, abstinence incentives, and an FAQ section. PWS participants also received one iCOquit® monitor [29], eight Alere™ iScreen® test kits, instructions to download and use the Vincere Health icoVita app [30], and check-in instructions. The family member also received printed evidence-based tips on how to support a loved one in quitting smoking, including quitting together as a supportive action. Because there is currently limited data on the impact of enrolling with a support person who also smokes, our 10.13039/100014144RCT plans to examine this further. Thus, all family members received the same tobacco cessation materials as the PWS, as well as information on supporting a loved one in quitting smoking.

### 2.5 Intervention

#### 2.5.1 Schedule

PWS participants were scheduled to complete six smoking status check-ins during the intervention: Weeks 1, 2, 3, 4, Month 3, and Month 6. This schedule was chosen to provide frequent reinforcement for early abstinence given that relapse often occurs soon after a quit attempt [31], and multiple studies have suggested that a 2-week initial smoking abstinence period decreases risk of relapse [[32], [33], [34], [35]]. PWS and family members were each paid $25 for every smoking status check-in completed by the PWS, regardless of whether the test results confirmed smoking abstinence.

#### 2.5.2 Smoking status check-ins

PWS participants created a password-protected profile in the Vincere Health icoVita app and web-based portal, both secure and HIPAA-compliant software programs [30], to complete smoking status assessments. Each check-in included three parts to confirm abstinence.

1) The iCOquit® monitor is validated to detect carbon monoxide (CO) for several hours, and is not confounded by nicotine replacement therapy (NRT), smokeless tobacco, or e-cigarettes [[36], [37]]. The iCOquit® connects via Bluetooth to the icoVita app on a smartphone or tablet. During breath tests, the app displays instructions to inhale, hold the breath, and exhale into the iCOquit® monitor for 15–20 s. The CO parts per million (PPM) result is shown on the screen and recorded in the app. Test data are automatically uploaded in the Vincere Health portal. A CO result of ≤6 ppm was considered abstinent for incentive eligibility [38].
2) The Alere™ iScreen® cotinine test detects nicotine use within the last 1–2 days, but can be confounded by use of NRTs, smokeless tobacco, or e-cigarettes [39]. Results are displayed within 10 min as either positive or negative for cotinine. Participants completed the test, took the same two photos as during post-consent screening (face with swab in the mouth; test result), and uploaded them in the secure “Chat” feature of the icoVita app which was accessible through the Vincere portal by study coordinators (AY, BT, AB).
3) PWS answered three yes/no self-report questions which were sent through the icoVita app “Chat” feature: 1) “Have you smoked any cigarettes (even a puff) in the last 7 days? Includes cigarillos and roll-your-own cigarettes”; 2) “Have you used other nicotine or tobacco products in the last 7 days? Includes cigars, e-cigarettes, Iqmik, or Copenhagen/chew”; and 3) “Have you used nicotine patches, gum, lozenges, or spray in the last 7 days?”. Contingency management studies standardly collect self-reports to measure smoking or tobacco use that may not be detected by biochemical verification [[40], [41]]. Additionally, because the cotinine test is confounded by other nicotine/tobacco products, PWS were asked to report NRT or other nicotine/tobacco use to contextualize results for incentive eligibility. For example, if a PWS had a positive cotinine result but also reported using NRTs or other nicotine/tobacco products, they were still eligible for an incentive if the CO breath test was negative and they did not report smoking cigarettes. Participants with a positive CO test were not eligible for an incentive, regardless of self-reported smoking abstinence or a negative cotinine test result.

#### 2.5.3 Orientation

Study staff completed an orientation call via phone or Zoom with PWS to ensure they knew how to complete smoking status check-ins, and confirmed they would be due to start the smoking status check-ins in one week. PWS were emailed or mailed a calendar with personalized check-in due dates, a checklist for each check-in, links to videos demonstrating the iCOquit® monitor and Alere™ iScreen® cotinine tests, and the study email and phone number in case of questions or problems. Study staff notified enrolled family members by text message that their PWS was due to start check-ins in one week.

#### 2.5.4 Financial incentives

At each smoking status check-in, both the PWS and their family member received financial incentives when the assessments confirmed PWS smoking abstinence. The incentive schedule followed an escalating reinforcement [[42], [43]] scheme: $50, $75, $100, $125, $175, $225. Thus, PWS and family members could each earn a total of up to $750. If a check-in was completed on-time but the results did not confirm abstinence, the PWS and family member each received $25 but did not receive additional incentives.

The incentive amounts began at $50 regardless of when the first abstinent result was achieved. The amounts increased with each subsequent abstinent check-in. If a PWS who had been abstinent later missed or tested positive for smoking at a check-in, the incentive amount for the next abstinent check-in was restored to the same value as the last incentive earned, and the escalation started again from that amount [31]. For example, if a PWS earned $50 at Week 1 and $75 at Week 2, did not earn an incentive at Week 3, but earned an incentive at Week 4, the amount earned at Week 4 was again $75. Dyad participants were notified via text message whether the check-in had been completed or missed. If the check-in was completed, both participants received an additional text message confirming the results and incentive amount, if earned.

Participants chose to receive payments as either prepaid cash cards or direct deposit. Participants interested in direct deposit were informed that payments could take up to four weeks, and completed an electronic transfer form with their bank account information to accept payments from the ANTHC.

### 2.6 End-of-study survey

At the end of the 6-month intervention, participants completed a follow-up survey online in REDCap or by phone. The survey assessed intervention acceptability including the perceived helpfulness of the intervention, whether participants would recommend the program to others, and open-ended questions on suggested changes or improvements to the program. The PWS survey additionally assessed self-reported smoking and other nicotine/tobacco use in the past seven days, average daily cigarettes smoked, quit attempts, use of cessation resources, perceived helpfulness of involving a family member in the program, and how the family member provided support. If the family member reported smoking at baseline, additional questions assessed self-reported cigarette smoking and other nicotine/tobacco use in the past seven days, average daily cigarettes smoked, quit attempts, and use of cessation resources. Participants received a $25 prepaid cash card for completing the end-of-study survey.

### 2.7 Statistical analyses

Screening data was summarized using descriptive statistics (percentages, means, ranges) to assess advertising reach and ineligibility reasons, and to describe the total numbers screened, eligible, and geographic location patterns of potential participants. Descriptive statistics were also used to assess study retention, engagement with the intervention, smoking behaviors, participant satisfaction, and technical challenges.

## 3 Results

### 3.1 Participants

Of the 100 potential PWS screened, 64 (64 %) were eligible. Of 43 screened potential family members, 36 (84 %) were eligible. Most participants were recruited through Facebook or family and friends. The most common reason for ineligibility among PWS was cessation pharmacotherapy use within the past 3 months; for family members, it was not having an eligible or interested PWS to enroll with. In total, 13 PWS consented and completed the Alere™ iScreen® screening test. One had a negative test and was not eligible, and two others failed to accrue with a family member before the study met its accrual goal. Fifteen family members consented, but five failed to accrue with a PWS. In total, 10 PWS and 10 family members were enrolled in the study.

See Table 2 for participant demographic characteristics. The PWS mean age was 45 years (range = 34–57), eight (80 %) were women, and six (60 %) lived outside of Anchorage. The family members' mean age was 30 years (range = 18–38; note: 5/10 reported age), five (50 %) were women, six (60 %) lived outside of Anchorage, nine (90 %) were ANAI, and four (40 %) were a PWS’ spouse or significant other. The mean cigarettes smoked per day for PWS was 13 (range = 3–20). Among family member participants, 40 % also smoked and reported smoking a mean of seven cigarettes per day (range = 4–10).

**Table 2:** Aniqsaaq beta-test participant characteristics.

	Person who Smokes (n = 10)	Family Members (n = 10)
**Age in years**		
Mean (range)	45 (34–57)	30 (18–38)^a^
**Gender**		
Female	8 (80 %)	5 (50 %)
**Race** (could choose more than one)		
ANAI	10 (100 %)	9 (90 %)
White	4 (40 %)	6 (60 %)
Hawaiian/Pacific Islander	1 (10 %)	–
**ANAI ethnicity** (could choose more than one)		
Aleut	1 (10 %)	2 (22 %)
Alutiiq	1 (10 %)	–
Athabaskan	3 (30 %)	3 (33 %)
Inupiat	3 (30 %)	3 (33 %)
Unanga $\hat{x}$	3 (30 %)	1 (11 %)
Yup'ik	4 (40 %)	3 (33 %)
**Marital status**		
Single	4 (40 %)	6 (60 %)
Divorced	1 (10 %)	–
Married/life partnership	5 (50 %)	4 (40 %)
**Rural residence** (live outside of Anchorage)	6 (60 %)	6 (60 %)
**Highest education**		
High school graduate/GED	2 (20 %)	7 (70 %)
Some college, no degree	2 (20 %)	2 (20 %)
Trade/technical school	1 (10 %)	–
Associate's or Bachelor's degree	5 (50 %)	1 (10 %)
**Currently employed for a paycheck**	6 (60 %)	5 (50 %)
**Lives with co-enrolled family member**	7 (70 %)	n/a
**Family member relationship type**	n/a	
Spouse/significant other		4 (40 %)
Child		3 (30 %)
Sibling		2 (20 %)
Distant relative		1 (10 %)
**Perceived closeness to co-enrolled family member (1=not close at all, 10=very close)**	n/a	
Mean (range)		9.3 (6–10)

a n = 5 family members provided age.

### 3.2 Engagement

**Fig. 1:**
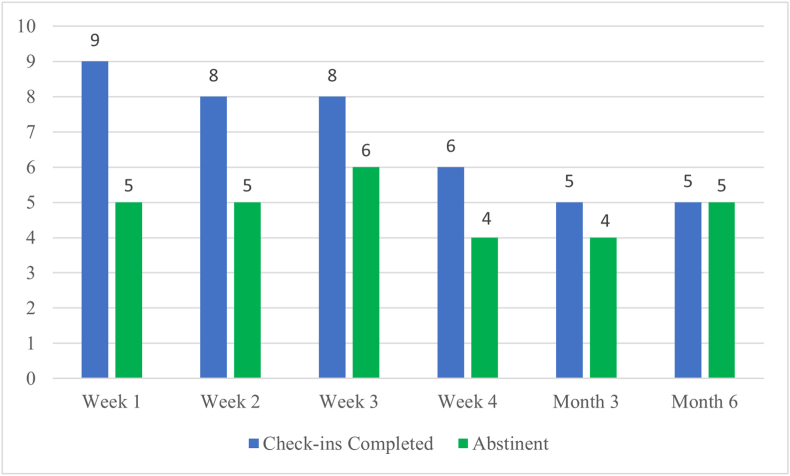
Phase 2 beta-test engagement and abstinence rates Legend: Completion and abstinence rates for all 10 PWS participants. Alt text: Bar graph showing the completion and abstinence rates for all 10 PWS participants.

Across the 10 PWS, there were 60 total check-ins possible over the 6-month intervention, of which 41 (68 %) were completed. Half of PWS (50 %) completed all six scheduled check-ins. Eight of 10 PWS (80 %) completed at least two check-ins, and seven of 10 (70 %) completed at least three check-ins. Fig. 1 shows the overall intervention engagement and abstinence rates.

### 3.3 Incentive payments

Seven PWS and six family members chose to receive incentives via direct deposit. The direct deposit set-up and initial payment typically took 3–4 weeks, while subsequent payments were issued within 1–2 weeks of a completed check-in. Prepaid cash cards were mailed to all other participants within two business days of completed check-ins. Nine dyads earned a total of $5450 in incentives for smoking abstinence during the 6-month intervention. An additional $2050 in remuneration was paid to the nine dyads for completing a total of 41 check-ins, regardless of abstinence.

### 3.4 Smoking abstinence

**Fig. 2:**
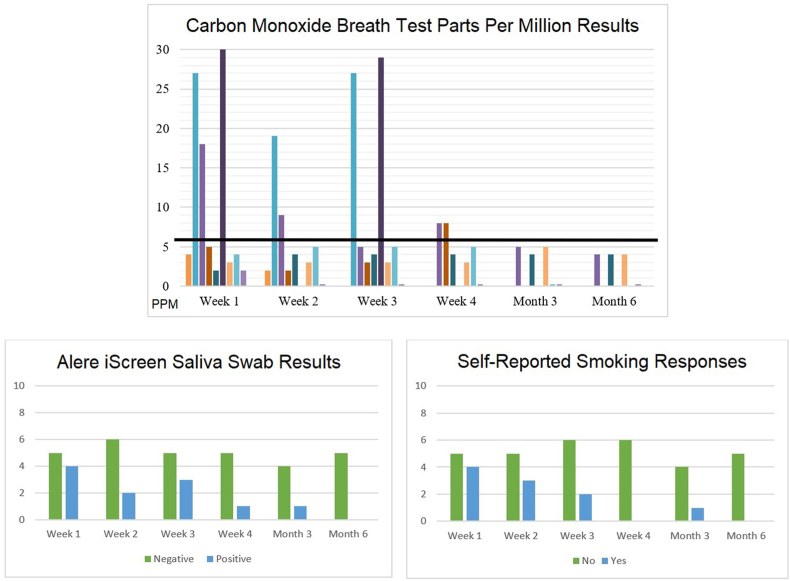
Smoking status results for each assessment type at all check-ins Legend: Carbon monoxide (CO) parts per million (PPM) results are shown for all 10 PWS participants. The black line at 6 PPM represents the cutoff for smoking abstinence results. Alere iScreen results are shown for all 10 PWS participants. Self-reported smoking response results are shown for all 10 PWS participants. Alt text: The first bar graph shows carbon monoxide parts per million (PPM) results for all 10 PWS participants. A black line depicts the 6 PPM cutoff for smoking abstinence results. The second bar graph shows Alere iScreen saliva swab test results, reported as positive or negative, for all 10 PWS participants. The third bar graph shows self-reported smoking response results, reported as yes or no, for all 10 PWS participants.

Two of 10 PWS (20 %) were abstinent at all check-ins and earned the $750 maximum amount possible. The five PWS who completed all check-ins were all abstinent at Month 6. Two participants had incentive amounts restored after positive test results. See Table 3 for individual check-in results. Of the 41 completed check-ins, 38 (93 %) demonstrated agreement between the iCOquit® monitor, Alere™ iScreen®, and self-report questions. Two PWS reported smoking cigarettes within the last seven days but had negative CO and cotinine tests. One PWS had a negative cotinine test and no reported cigarette smoking but had a CO result of 8 PPM. See Fig. 2 for results at each check-in.

**Table 3:**
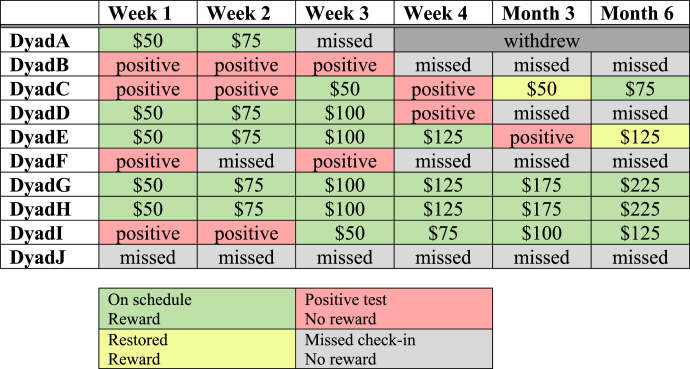
Check-in results and incentives earned by Phase 2 beta-test participants.

### 3.5 NRT and other nicotine or tobacco use

Half of PWS (5/10) reported NRT use during the 6-month intervention: two reported use at one check-in, two reported use at three check-ins, and one reported use at four check-ins. Of note, all five participants who reported NRT use were the only participants to complete the final check-in, and all were abstinent at Month 6. One PWS reported using other nicotine or tobacco products at two check-ins. On the baseline and end-of-study surveys, no PWS or family members reported using other nicotine or tobacco products.

### 3.6 End-of-study survey

*People Who Smoke:* Half of PWS (5/10) completed the end-of-study survey. Four of the five reported not smoking any cigarettes in the last seven days, and three of the five reported using cessation resources recommended in study materials (Alaska's State Quitline, healthcare provider). One PWS had completed half of the study check-ins, and reported that they had not tried to quit and were still smoking.

Regarding acceptability, four of the five PWS rated the family-based incentives program as “very helpful” in quitting smoking, rated having their family member in the program with them as “very helpful”, and “definitely would” recommend the program to other Alaska Native people interested in quitting. Four PWS reported that it was helpful to have family members “encourage them to keep going” and “celebrate their successes”. When asked to identify positive aspects of the program, all five PWS selected “Financial rewards” from a list of options. One PWS reported, “I've tried to quit a lot of times over the years. The monetary incentives helped me.” and another shared, “It helped me quit in 6 months' time!!” Other positive aspects selected included: biochemical monitoring, family support, smoking cessation treatment resources, and encouraging text messages. Four PWS noted that delays in receiving incentives or shipped materials were a negative aspect, and one reported Wi-Fi and data connection problems.

*Family members:* Three of 10 family members completed the end-of-study survey. All three felt that the program was “somewhat” or “very helpful” to support their family member. Responses varied from “probably not” to “definitely” would recommend the program to other family members. Positive aspects selected from a list of options included: rewards, smoking status check-ins, family support resources, and cessation treatment resources.

### 3.7 Technical challenges

PWS demonstrated understanding of the app and test devices during the orientation call and none reported experiencing problems that prevented them from completing check-ins. One PWS withdrew from the study after Week 3, and one did not complete any check-ins despite contact by the study team. One participant reported disconnecting from the internet before test photos had finished uploading. The study team asked them to email the timestamped photos and confirmed the tests had been completed during the allowed window. One participant lost a test kit before the 6-month check-in, was mailed a replacement, and had the completion window extended by three days to allow for shipping.

Technical difficulties with the Vincere Health portal and app included: study staff login challenges, photos to confirm participant identity were unavailable for half of all check-ins, and some participants had difficulty uploading cotinine test photos in the app. Additionally, PWS received a text message at each check-in with a link to answer the three self-reported questions. However, the initial wording was confusing (“You've been selected for a short survey!”) and led early participants to delete the link. The wording was changed later, and the questions were also available through the icoVita app.

Though all participants received the incentives they earned, there were unexpected payment delays. The set-up for direct deposit payments was lengthier than expected and meant that incentive payments could not provide immediate reinforcement [44]. Participants who enrolled later were informed that cash cards could be issued sooner, but several still chose direct deposit. At one point, study staff had difficulty ordering prepaid cash cards which meant some payments were delayed by a few weeks. Some participants also had difficulty receiving the prepaid cash cards by mail due to uncommunicated address changes or delayed post office box pick-up.

## 4 Discussion

The Aniqsaaq study was co-developed through a CBPR approach with Alaska ANAI families, healthcare providers, and researchers. Adaptations were made to the study design and materials based on Phase 1 interview feedback. The present beta-test demonstrated that a novel, culturally tailored, remote financial incentive intervention with ANAI families was feasible with respect to recruitment, engagement, and intervention acceptability.

Some prior studies reported on dyadic adaptations of financial incentive interventions have been shown to be feasible and have promising effects on smoking cessation. These enrolled dyads of dual-smoker couples [45], pregnant women who smoked and a female non-smoking support person [46], and pregnant women with an optional enrollment of a family member regardless of smoking status [47]. An 10.13039/100014144RCT enrolled women-child dyads to promote maternal smoking abstinence and reduce child second-hand smoke exposure [48]. In another study enrolling adults from rural villages in Thailand, participants were assigned or self-selected to join with a teammate who also smoked [49]. Both members of dyad teams were more likely to quit smoking when self-selected compared to randomly assigned teams (27 % vs. 4 %, p < 0.001), highlighting the value of naturally occurring social support networks in quitting. To the best of our knowledge, financial incentive cessation interventions focused on the family dyad have not been evaluated among ANAI communities.

Engagement with the 6-month intervention was high, with 90 % of PWS participants completing at least two check-ins, and 50 % completing all six check-ins. Most participants were able to submit at-home test results without difficulty. Challenges included personal Wi-Fi connection issues, technical difficulties with the icoVita app, and delays in receiving incentive payments. However, all participants were able to complete check-ins and received payments, and improvements were made for the planned RCT. Though participants were ineligible if they reported cessation treatment in the past three months, evidence-based cessation treatments were recommended after enrollment. Encouragingly, half of PWS reported using NRTs, and all were abstinent at the final check-in. The RCT will further explore intervention effects on self-reported cessation treatment utilization. In addition, the RCT will explore other smoking-related outcomes among PWS, such as reductions in daily cigarettes smoked and other tobacco and nicotine product use.

Among family member participants, four reported current smoking at the time of enrollment. Further exploration of the impact of co-enrolling with a family member who also smokes will be assessed in the RCT, as well as changes in smoking-related behaviors among family members.

The end-of-study survey responses indicated that participants generally had a positive experience with the intervention. PWS survey respondents identified the incentives as a positive component of the intervention, and most felt that having a family member in the program was helpful. However, further exploration on family support is needed because only half of PWS completed the survey, a response rate which is consistent with prior research [[50], [51], [52]]. Overall, the PWS engagement and 6-month abstinence rates showed a promising signal toward efficacy consistent with other financial incentive intervention studies [[53], [54]].

### 4.1 Adaptations for the RCT

We shared our results with the ANTHC RCC which had positive feedback on the beta-test findings and our planned improvements for the RCT. Because PWS were able to complete at-home tests and upload photos to REDCap during screening, all study procedures for the RCT will be completed in REDCap: PWS participants will upload test photos and answer self-report questions on one page that includes embedded how-to videos for the testing devices. The calls to review the consent form and check-in orientation will be offered optionally. The “resources” provided by the study will be described as “informational materials” to clarify that although use of evidence-based smoking cessation treatments including NRTs, medications, or counseling are encouraged, they are not provided directly by the study. In line with organizational processes for research participant remuneration, RCT incentives will be provided through either a prepaid cash card, or a virtual ClinCard® that can be added to a digital wallet or used online typically within 1 h of the study team issuing the payment.

### 4.2 Strengths and limitations

There were several strengths of the present Phase 2 beta-test. Because novel studies often encounter logistical challenges at the start, beta-testing allows study teams to evaluate processes and materials in the real-world. While the sample size was necessarily small, we enrolled a variety of family relationships, ANAI cultures, and statewide locations. Furthermore, incorporating lessons learned throughout the CBPR-informed development of subsequent phases is more efficient than experiencing challenges and delays at the start of a large trial. While breath CO detects only recent smoking, we also used saliva cotinine to verify abstinence, and there was good agreement between biochemical test results and self-reports.

Our beta-test also had limitations. The mean PWS age of 45 years and range of 34–57 years did not allow us to get feedback from young adults who may have different smoking patterns or preferences for at-home tests and incentive payments, or from older adults who may have different comfort levels with technology. Some processes will be adjusted for the RCT, and these new procedures will not have been beta-tested (e.g., randomization, ClinCard®). Additionally, beta-test participants may have felt their participation was complete after the intervention ended, with only 50 % of PWS and 30 % of family members completing the end-of-study survey. Follow-up on the RCT will continue for one year after the intervention ends, and participants will continue to receive payments for completing remote check-ins and surveys.

## 5 Conclusions and future directions

The current beta-test supported the feasibility of an ANAI family-based financial incentive intervention. This intervention will be evaluated for effectiveness in an RCT enrolling 656 PWS-family member dyads. We will also conduct qualitative interviews with PWS, family members, and Alaska Tribal Health System stakeholders, as well as a cost-effectiveness analysis, to inform long-term statewide implementation.

Future clinical implementers may consider alternative incentive types recommended by Phase 1 participants (e.g., gas cards, grocery vouchers, winter gear, etc.) [21] which are difficult to offer in large research trials. Moreover, future iterations of the intervention might consider providing contingencies for reducing smoking to reinforce successive approximations toward abstinence, as successfully implemented in prior financial incentive interventions [55].

## CRediT authorship contribution statement

**Brianna N. Tranby:** Writing – review & editing, Writing – original draft, Visualization, Validation, Resources, Project administration, Investigation, Data curation. **Antonia M. Young:** Writing – review & editing, Writing – original draft, Visualization, Resources, Project administration, Investigation, Data curation. **Anne I. Roche:** Writing – review & editing, Writing – original draft, Data curation. **Flora R. Lee:** Writing – review & editing, Resources, Project administration, Investigation, Data curation. **Ashley R. Brown:** Writing – review & editing, Resources, Project administration, Investigation, Data curation. **Barb J. Stillwater:** Writing – review & editing, Supervision, Resources, Project administration. **Judith J. Prochaska:** Writing – review & editing, Methodology, Funding acquisition, Conceptualization. **Diane K. King:** Writing – review & editing, Funding acquisition, Conceptualization. **Paul A. Decker:** Writing – review & editing, Methodology, Funding acquisition. **Bijan J. Borah:** Writing – review & editing, Funding acquisition, Conceptualization. **Michael G. McDonell:** Writing – review & editing, Funding acquisition, Conceptualization. **Timothy K. Thomas:** Writing – review & editing, Supervision, Resources, Project administration, Methodology, Investigation, Funding acquisition, Conceptualization. **Christi A. Patten:** Writing – review & editing, Supervision, Resources, Project administration, Methodology, Investigation, Funding acquisition, Conceptualization.

## Data availability

On reasonable request to the corresponding author, the data underlying this article will be shared after any necessary Tribal committee approval.

## Funding

This work was supported by the National Institute on Drug Abuse (NIDA) of the National Institutes of Health [grant number R01 DA046008] received by CP. The content is solely the responsibility of the authors and does not necessarily represent the official views of the National Institutes of Health.

## Declaration of competing interest

The authors declare the following financial interests/personal relationships which may be considered as potential competing interests:Unrelated to this project, Dr. Prochaska has provided consultation to pharmaceutical and technology companies that make medications and other treatments for quitting smoking. Dr. Prochaska has also served as an expert witness in lawsuits against tobacco companies. The other authors report no potential conflicts of interest.

## Data Availability

Data will be made available on request.

## References

[1] Alaska Department of Health Division of Public Health, Section of Chronic Disease Prevention and Health Promotion. Alaska Tobacco Facts: 2023 Update 2023. Available from: https://health.alaska.gov/dph/Chronic/Documents/Tobacco/PDF/2023_AKTobaccoFacts.pdf.

[2] Mowery P.D., Dube S.R., Thorne S.L., Garrett B.E., Homa D.M., Nez Henderson P. (2015). Disparities in smoking-related mortality among American Indians/Alaska natives. Am. J. Prev. Med..

[3] Nash S.H., Day G., Zimpelman G., Hiratsuka V.Y., Koller K.R. (2019). Cancer incidence and associations with known risk and protective factors: the Alaska EARTH study. Cancer Causes Control.

[4] Fortuine R. (1996). Historical notes on the introduction of tobacco into Alaska. Alaska Med..

[5] Redwood D., Lanier A.P., Renner C., Smith J., Tom-Orme L., Slattery M.L. (2010). Differences in cigarette and smokeless tobacco use among American Indian and Alaska Native people living in Alaska and the Southwest United States. Nicotine Tob. Res..

[6] Renner C.C., Patten C.A., Enoch C., Petraitis J., Offord K.P., Angstman S., Garrison A., Nevak C., Croghan I.T., Hurt R.D. (2004). Focus groups of Y-K Delta Alaska Natives: attitudes toward tobacco use and tobacco dependence interventions. Prev. Med..

[7] Patten C.A., Hiratsuka V.Y., Nash S.H., Day G., Redwood D.G., Beans J.A., Howard B.V., Umans J.G., Koller K.R. (2022). Smoking patterns among urban Alaska native and American Indian adults: the Alaska EARTH 10-year follow-up study. Nicotine Tob. Res..

[8] Alaska Department of Health and Social Services and the Alaska Native Tribal Health Consortium (2019). https://www.healthyalaskans.org/wp-content/uploads/2020/03/HA2030_StateHealthAssessment.pdf.

[9] Walch A.K., Ohle K.A., Koller K.R., Alexie L., Lee F., Palmer L., Nu J., Thomas T.K., Bersamin A. (2022). Impact of assistance programs on indigenous ways of life in 12 rural remote western Alaska native communities: elder perspectives shared in formative work for the "got neqpiaq?" Project. Int. J. Circumpolar Health.

[10] Maddox R., Drummond A., Kennedy M., Martinez S.A., Waa A., Nez Henderson P., Clark H., Upton P., Lee J.P., Hardy B.-J., Tautolo E.-S., Bradbrook S., Calma T., Whop L.J. (2023). Ethical publishing in ‘Indigenous’ contexts. Tob. Control.

[11] Ladapo J.A., Prochaska J.J. (2016). Paying smokers to quit: does it work? Should we do it?. J. Am. Coll. Cardiol..

[12] Etter J.F., Schmid F. (2016). Effects of large financial incentives for long-term smoking cessation: a randomized trial. J. Am. Coll. Cardiol..

[13] Halpern S.D., French B., Small D.S., Saulsgiver K., Harhay M.O., Audrain-McGovern J., Loewenstein G., Brennan T.A., Asch D.A., Volpp K.G. (2015). Randomized trial of four financial-incentive programs for smoking cessation. N. Engl. J. Med..

[14] Notley C., Gentry S., Livingstone-Banks J., Bauld L., Perera R., Conde M., Hartmann-Boyce J. (2025). Incentives for smoking cessation. Cochrane Database Syst. Rev..

[15] Patten C.A., Fadahunsi O., Hanza M.M., Smith C.A., Decker P.A., Boyer R., Ellsworth L., Brockman T.A., Hughes C.A., Bronars C.A., Offord K.P. (2014). Tobacco cessation treatment for Alaska native adolescents: group randomized pilot trial. Nicotine Tob. Res..

[16] Koller K.R., Flanagan C.A., Day G.E., Thomas T.K., Smith C.A., Wolfe A.W., Meade C., Hughes C.A., Hiratsuka V.Y., Murphy N.J., Patten C.A. (2017). Developing a biomarker feedback intervention to motivate smoking cessation during pregnancy: phase II MAW study. Nicotine Tob. Res..

[17] Hirchak K.A., Leickly E., Herron J., Shaw J., Skalisky J., Dirks L.G., Avey J.P., McPherson S., Nepom J., Donovan D., Buchwald D., McDonell M.G. (2018). Focus groups to increase the cultural acceptability of a contingency management intervention for American Indian and Alaska Native Communities. J. Subst. Abuse Treat..

[18] Anderson K.M., Kegler M.C., Bundy L.T., Henderson P., Halfacre J., Escoffery C. (2019). Adaptation of a brief smoke-free homes intervention for American Indian and Alaska Native families. BMC Public Health.

[19] Merculieff Z.T., Koller K.R., Sinicrope P.S., Hughes C.A., Bock M.J., Decker P.A., Resnicow K., Flanagan C.A., Meade C.D., McConnell C.R., Prochaska J.J., Thomas T.K., Patten C.A. (2020). Developing a social media intervention to connect Alaska native people who smoke with resources and support to quit smoking: the connecting Alaska native quit study. Nicotine Tob. Res..

[20] Carroll D.M., Jennings D., Stately A., Kamath A., Tessier K.M., Cotoc C., Egbert A., Begnaud A., Businelle M., Hatsukami D., Pickner W. (2024). Pilot randomised controlled trial of a culturally aligned smoking cessation app for American Indian persons. Tob. Control.

[21] Patten C.A., Smith C.M., Brockman T.A., Decker P.A., Hughes C.A., Nadeau A.M., Sinicrope P.S., Offord K.P., Lichtenstein E., Zhu S.H. (2011). Support-person promotion of a smoking quitline: a randomized controlled trial. Am. J. Prev. Med..

[22] Scholz U., Stadler G., Ochsner S., Rackow P., Hornung R., Knoll N. (2016). Examining the relationship between daily changes in support and smoking around a self-set quit date. Health Psychol..

[23] Thomas D.P., Davey M.E., van der Sterren A.E., Lyons L., Hunt J.M., Bennet P.T. (2019). Social networks and quitting in a national cohort of Australian Aboriginal and Torres Strait Islander smokers. Drug Alcohol Rev..

[24] Patten C.A., Koller K.R., King D.K., Prochaska J.J., Sinicrope P.S., McDonell M.G., Decker P.A., Lee F.R., Fosi J.K., Young A.M., Sabaque C.V., Brown A.R., Borah B.J., Thomas T.K. (2023). Aniqsaaq (To Breathe): study protocol to develop and evaluate an Alaska Native family-based financial incentive intervention for smoking cessation. Contemp Clin Trials Commun.

[25] Dillard D.A., Caindec K., Dirks L.G., Hiratsuka V.Y. (2018). Challenges in engaging and disseminating health research results among Alaska native and American Indian people in southcentral Alaska. Am. Indian Alsk. Native Ment. Health Res..

[26] Springer M.V., Skolarus L.E. (2019). Community-based participatory research. Stroke.

[27] Sinicrope P.S., Tranby B.N., Young A.M., Koller K.R., King D.K., Lee F.R., Sabaque C.V., Prochaska J.J., Borah B.J., Decker P.A., McDonell M.G., Stillwater B., Thomas T.K., Patten C.A. (2024). Adapting a financial incentives intervention for smoking cessation with Alaska native families: phase 1 qualitative research to inform the Aniqsaaq (to breathe) study. Nicotine Tob. Res..

[28] Rounsaville B.J., Carroll K.M., Onken L.S. (2001). A stage model of behavioral therapies research: getting started and moving on from stage I. Clin. Psychol. Sci. Pract..

[29] Baker T.B., Gustafson D.H., Shah D. (2014). How can research keep up with eHealth? Ten strategies for increasing the timeliness and usefulness of eHealth research. J. Med. Internet Res..

[30] Nielsen J. (2012). How many test users in a usability study?. https://www.nngroup.com/articles/how-many-test-users/.

[31] Faseru B., Richter K.P., Scheuermann T.S., Park E.W. (2018). Enhancing partner support to improve smoking cessation. Cochrane Database Syst. Rev..

[32] Winter N.B., Tyler Kennedy, Ryan, Clifford Scott (2019). A simplified protocol to screen out VPS and international respondents using Qualtrics. https://ssrn.com/abstract=3327274.

[33] Moore M.R., Mason M.J., Brown A.R., Garcia C.M., Seibers A.D., Stephens C.J. (2018). Remote biochemical verification of tobacco use: reducing costs and improving methodological rigor with mailed oral cotinine swabs. Addict. Behav..

[34] Gachter S., Starmer C., Tufano F. (2015). Measuring the closeness of relationships: a comprehensive evaluation of the 'inclusion of the other in the self' scale. PLoS One.

[35] iCOquit (2020). About the iCOquit(R). https://www.icoquit.com/us/.

[36] Vincere Health. icoVita App [cited July 14, 2024]. Available from: https://www.vincere.health/icovita-privacy-policy.

[37] Connolly T., Butler David (2006). Regret in economic and psychological theories of choice. J. Behav. Decis. Making.

[38] Alessi S.M., Badger G.J., Higgins S.T. (2004). An experimental examination of the initial weeks of abstinence in cigarette smokers. Exp. Clin. Psychopharmacol.

[39] Bradstreet M.P., Higgins S.T., McClernon F.J., Kozink R.V., Skelly J.M., Washio Y., Lopez A.A., Parry M.A. (2014). Examining the effects of initial smoking abstinence on response to smoking-related stimuli and response inhibition in a human laboratory model. Psychopharmacology (Berl)..

[40] Kenford S.L., Fiore M.C., Jorenby D.E., Smith S.S., Wetter D., Baker T.B. (1994). Predicting smoking cessation. Who will quit with and without the nicotine patch. JAMA.

[41] Romanowich P., Lamb R.J. (2010). The relationship between in-treatment abstinence and post-treatment abstinence in a smoking cessation treatment. Exp. Clin. Psychopharmacol.

[42] Ramani V.K., Mhaske M., Naik R. (2023). Assessment of carbon monoxide in exhaled breath using the smokerlyzer handheld machine: a cross- sectional study. Tob. Use Insights.

[43] bedfont (2025). iCOquit® Smokerlyzer® FAQ. https://www.icoquit.com/us/faq/#1586190438068-03090fc4-8133.

[44] Middleton E.T., Morice A.H. (2000). Breath carbon monoxide as an indication of smoking habit. Chest.

[45] Alere. Nicotine Testing – Common Questions 2012 [July 16, 2024]. Available from: https://www.newlinemedical.com/assets/pdf/Alere-iScreen-Cotinine-FAQ.pdf.

[46] Rash C.J., Petry N.M., Alessi S.M. (2018). A randomized trial of contingency management for smoking cessation in the homeless. Psychol. Addict. Behav..

[47] Molina M.F., Hall S.M., Stitzer M., Kushel M., Chakravarty D., Vijayaraghavan M. (2022). Contingency management to promote smoking cessation in people experiencing homelessness: leveraging the electronic health record in a pilot, pragmatic randomized controlled trial. PLoS One.

[48] Roll J.M., Higgins S.T., Badger G.J. (1996). An experimental comparison of three different schedules of reinforcement of drug abstinence using cigarette smoking as an exemplar. J. Appl. Behav. Anal..

[49] Lamb R.J., Kirby K.C., Morral A.R., Galbicka G., Iguchi M.Y. (2010). Shaping smoking cessation in hard-to-treat smokers. J. Consult. Clin. Psychol..

[50] Critchfield T.S., Kollins S.H. (2001). Temporal discounting: basic research and the analysis of socially important behavior. J. Appl. Behav. Anal..

[51] vanDellen M.R., Wright J.W.C., Zhao B., Cullinan C., Beach S.R.H., Shen Y., Haskins L.B., Schiavone W.M., MacKillop J.M. (2024). Partner-involved financial incentives for smoking cessation in dual-smoker couples: a randomized pilot trial. Nicotine Tob. Res..

[52] Donatelle R.J., Prows S.L., Champeau D., Hudson D. (2000). Randomised controlled trial using social support and financial incentives for high risk pregnant smokers: significant other supporter (SOS) program. Tob. Control.

[53] Wen X., Eiden R.D., Justicia-Linde F.E., Wang Y., Higgins S.T., Thor N., Haghdel A., Peters A.R., Epstein L.H. (2018). A multicomponent behavioral intervention for smoking cessation during pregnancy: a nonconcurrent multiple-baseline design. Translational Behavioral Medicine.

[54] Higgins S.T., Plucinski S., Orr E., Nighbor T.D., Coleman S.R.M., Skelly J., DeSarno M., Bunn J. (2023). Randomized clinical trial examining financial incentives for smoking cessation among mothers of young children and possible impacts on child secondhand smoke exposure. Prev. Med..

[55] White J.S., Dow W.H., Rungruanghiranya S. (2013). Commitment contracts and team incentives: a randomized controlled trial for smoking cessation in Thailand. Am. J. Prev. Med..

